# CircRNA circ-PDCD11 promotes triple-negative breast cancer progression via enhancing aerobic glycolysis

**DOI:** 10.1038/s41420-021-00604-y

**Published:** 2021-08-21

**Authors:** Zeyu Xing, Ruojiao Wang, Xin Wang, Jiaqi Liu, Menglu Zhang, Kexin Feng, Xiang Wang

**Affiliations:** 1 National Cancer Center/National Clinical Research Center for Cancer/Cancer Hospital, Chinese Academy of Medical Sciences and Peking Union Medical College, Beijing, 100021 China; 2 Department of Medical Ultrasound, Peking Union Medical College Hospital, Chinese Academy of Medical Science and Peking Union Medical College, Beijing, 100730 China

**Keywords:** Cancer epigenetics, Oncogenesis

## Abstract

Well-described evidence has demonstrated the critical roles of aerobic glycolysis in triple-negative breast cancer (TNBC) oncotherapy. Moreover, next-generation high-throughput sequencing indicates the potential regulation of energy metabolism by circular RNAs (circRNAs) in TNBC. However, circRNA modulation of TNBC aerobic glycolysis is still unclear. Here, the present research aimed to investigate the function and underlying mechanisms of novel circPDCD11 (hsa_circ_0019853) in TNBC aerobic glycolysis. The results revealed that circPDCD11 expression was significantly upregulated in TNBC tissues and cells. Clinical data demonstrated that the high expression of circPDCD11 was closely correlated with a poor prognosis and acted as an independent risk factor for TNBC prognosis. Functionally, in vitro gain- and loss-of-function experiments revealed that circPDCD11 accelerated glucose uptake, lactate production, ATP generation, and the extracellular acidification rate in TNBC cells. In vivo, circPDCD11 silencing repressed tumor growth. Mechanistically, circPDCD11 acted as a miRNA sponge to enhance LDHA expression by sponging miR-432-5p. In conclusion, these combined results demonstrated that circPDCD11 acts as an oncogene for TNBC, providing a promising prognostic biomarker for TNBC.

## Introduction

Breast cancer is a progressive malignancy that is the leading cause of cancer-related mortality in women worldwide [1]. As a subtype, triple-negative breast cancer (TNBC) is emerging as one of the deadliest types of breast cancer. TNBC is characterized by the negative expression of estrogen receptor (ER), progesterone receptor (PR), and human epidermal growth factor receptor-2 (HER2) [2, 3]. Although great efforts have been made in etiological tracing research, limited progress has been achieved due to imprecise targeted treatments [4, 5]. To develop more effective targeted therapeutic strategies, more accurate pathogenesis initiators should be identified.

Circular RNAs (circRNAs) are a group of single-stranded noncoding RNAs derived from the corresponding gene elements by backsplicing, including introns, exons and intergenic regions [6–8]. Unlike linear RNAs, which are sensitive to RNA enzymes, circRNAs can resist degradation. In TNBC, the expression of a novel circRNA, circUBE2D2, is remarkably elevated, and its high expression is associated with advanced clinicopathologic features. CircUBE2D2 silencing reduces the doxorubicin resistance of TNBC cells by regulating the miR-512-3p/CDCA3 axis [9]. Moreover, circZEB1 is markedly overexpressed in TNBC tumor tissues and cell lines, thereby promoting proliferation and reducing cellular apoptosis through miR-448/eEF2K [10]. All this evidence suggests that circRNAs play an important role in TNBC oncogenesis.

Aerobic glycolysis, also known as the Warburg effect, is a crucial reprogrammed energy metabolism that is a hallmark of cancer. In TNBC, aerobic glycolysis has been shown to be an important carcinogen. In the present research, we assessed the circRNA expression profile in TNBC and identified a remarkably increased circRNA (circPDCD11, hsa_circ_0019853, circBase) in TNBC. The high expression of circPDCD11 was closely correlated with the unfavorable prognosis of TNBC patients. Functionally, circPDCD11 promoted the proliferation and aerobic glycolysis of TNBC cells through the miR-432-5p/LDHA axis. Our findings could highlight the critical role of circPDCD11 in TNBC, which may provide a potential therapeutic target for TNBC.

## Results

### CircRNA microarray demonstrated the high expression of circPDCD11 in TNBC

**Fig. 1:**
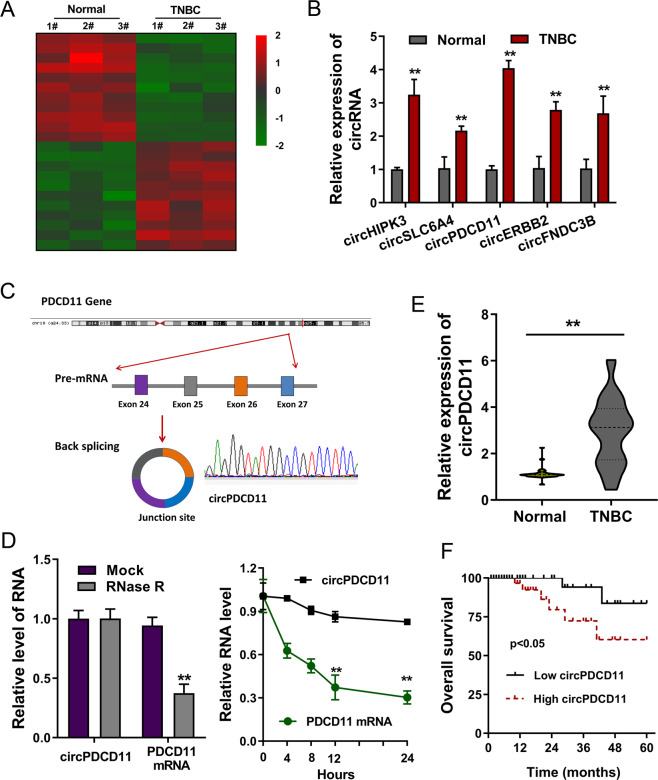
A circRNA microarray revealed the high expression of circPDCD11 in TNBC. **A** Heatmap of microarray analysis showing the up- and downregulated circRNAs in three pairs of cancer tissue and normal tissue. **B** RT-PCR demonstrated several upregulated circRNAs in TNBC specimens, including circPDCD11. **C** Schematic diagram showing that circPDCD11 was derived from PDCD11 pre-mRNA and generated through exon 27 ~ exon 24 back splicing (hsa_circ_0019853, 461 bp). **D** RT-PCR demonstrated circPDCD11 and linear transcript (PDCD11 mRNA) expression after treatment with RNase and actinomycin **D**. **E** RT-PCR demonstrated circPDCD11 expression in TNBC clinical specimens (32 samples) compared with adjacent normal specimens (32 samples). **F** Kaplan–Meier survival analysis illustrated the correlations between circPDCD11 expression (high = 16, low = 16) and overall survival of the TNBC patients from the enrolled samples. A p value was calculated using a log-rank test. Data are expressed as the mean ± SD. **p < 0.01.

To investigate the circRNA expression profile in TNBC, our team performed microarray analysis using three pairs of tissues (Fig. 1A). Numerous circRNAs were found to be dysregulated in TNBC tissue and normal tissue. Upon further investigation, RT-qPCR demonstrated that several circRNAs were upregulated in the TNBC specimens, including circPDCD11 (Fig. 1B). CircPDCD11 (hsa_circ_0019853, 461 bp) was derived from PDCD11 pre-mRNA and generated via backsplicing of exon 27 to exon 24 (Fig. 1C). Regarding the stability of circPDCD11, RNase and actinomycin D treatment assays demonstrated that circPDCD11 was more stable than the linear transcript upon extraneous treatment (Fig. 1D). In TNBC clinical specimens, we observed that circPDCD11 was significantly upregulated compared to that in adjacent normal specimens (Fig. 1E). Survival analysis (Kaplan–Meier) illustrated that high circPDCD11 expression in TNBC tissues was significantly associated with a worse overall survival (OS) (log-rank test, Fig. 1F, Table 1). Therefore, these results indicated that the high expression of circPDCD11 might be predictive for an unfavorable TNBC prognosis.

**Table 1:** Relationship between circPDCD11 and TNBC patients’ clinicopathological characteristic.

Characteristic		circPDCD11		*p*
		Low	High	
*Age*				
<50	19	8	11	0.341
≥50	13	8	5	
*Menopause*				
No	17	7	10	0.762
Yes	15	9	6	
*Tumor size*				
<2 cm	12	9	3	0.008*
≥2 cm	20	7	13	
*Distant metastasis*				
Negative	15	6	9	0.358
Positive	17	10	7	
*TNM*				
I, II	21	7	14	0.006*
III, IV	11	9	2	

*p < 0.05.

### circPDCD11 enhanced aerobic glycolysis in TNBC cells

**Fig. 2:**
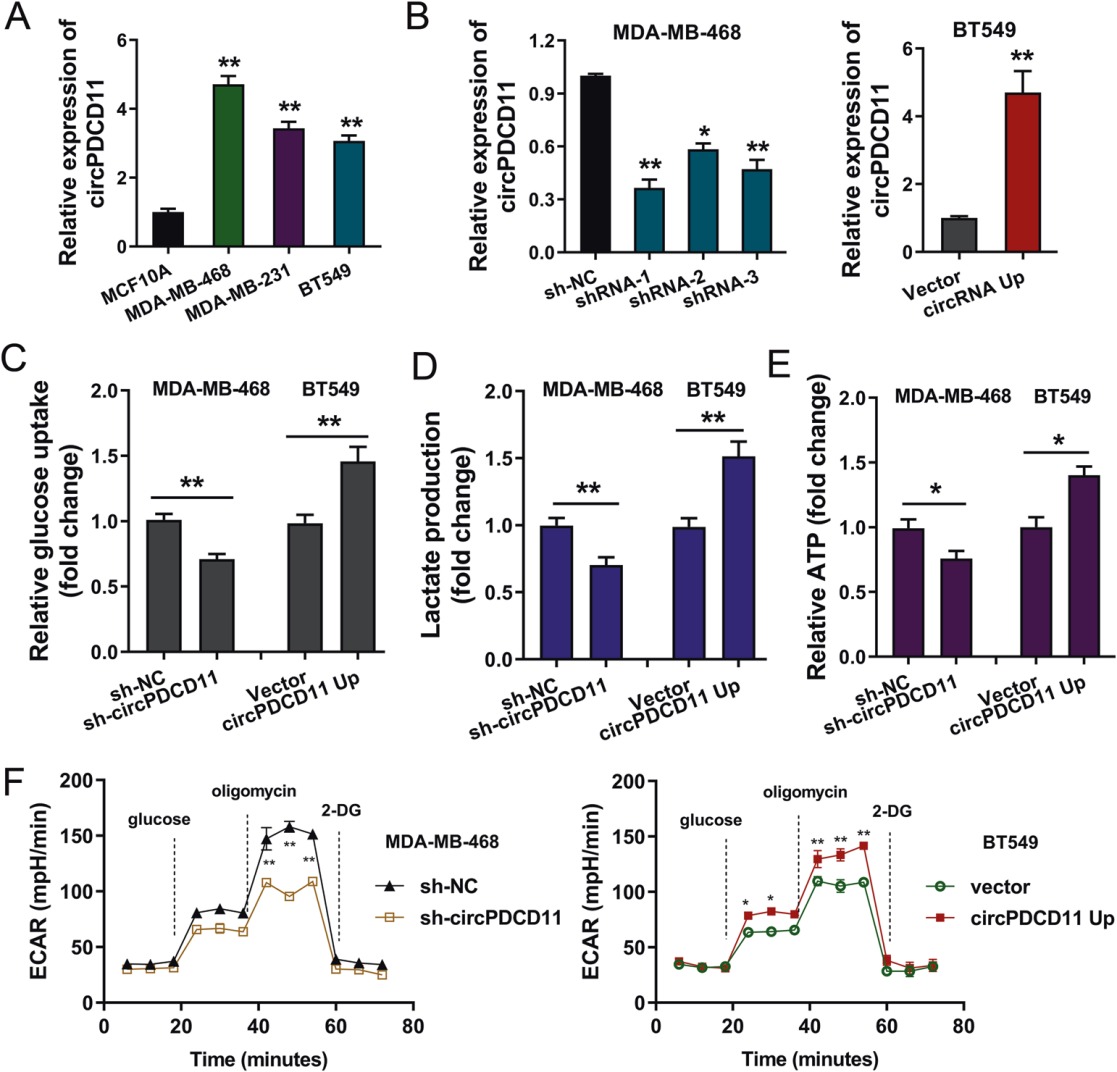
circPDCD11 enhanced aerobic glycolysis in TNBC cells. **A** RT-qPCR demonstrated circPDCD11 expression as measured in TNBC cell lines (BT549, MDA-MB-468, and MDA-MB-231) and human normal breast cells (MCF10A). **B** Lentivirus-mediated small-hairpin RNA (shRNA) and plasmid transfection were performed to effectively downregulate (MDA-MB-468) or upregulate (BT549) the endogenous expression of circPDCD11. **C** Glucose uptake was assessed using the Glucose Uptake Colorimetric Assay Kit. **D** Lactate-production analysis was quantified using a Lactate Colorimetric Assay Kit. **E** ATP quantity was quantified using the CellTiter-Glo Luminescent Cell Viability Assay Kit. **F** The extracellular acidification rate (ECAR) was quantified using a Bioscience XF96 Extracellular Flux Analyzer. The data are presented as the mean ± SD. Asterisks indicate significant differences (**p < 0.01, *p < 0.05).

To investigate the expression pattern of circPDCD11 in TNBC, in vitro functional assays and epigenetic experiments were performed. First, the expression of circPDCD11 was found to be highly expressed in TNBC cells compared to normal cells, as assessed by RT-PCR (Fig. 2A). Then, the expression of circPDCD11 was effectively knocked down in MDA-MB-468 cells through lentivirus-mediated small-hairpin RNA (shRNA) transfection (Fig. 2B, left). In addition, the expression of circPDCD11 was significantly upregulated in BT549 cells through plasmid transfection (Fig. 2B, right). In terms of energy metabolism, functional assays were performed. Loss-of-function assays revealed that the circPDCD11 knockdown significantly repressed glucose uptake (Fig. 2C), lactate production (Fig. 2D), ATP quantity (Fig. 2E), and the extracellular acidification rate (ECAR) (Fig. 2F) in MDA-MB-468 cells. In addition, gain-of-function assays revealed that circPDCD11 overexpression vitally promoted glucose uptake, lactate production, ATP quantity, and ECAR in BT549 cells. The above results indicated that upregulated circPDCD11 enhanced aerobic glycolysis in TNBC cells.

### circPDCD11 enhanced proliferation, and circPDCD11 silencing inhibited tumor growth

**Fig. 3:**
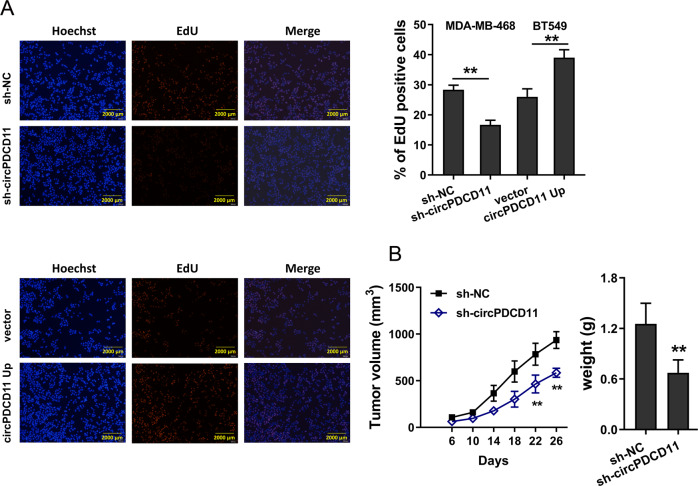
circPDCD11 enhanced proliferation, and its silencing inhibited tumor growth in vivo. **A** An EdU assay was performed to detect the proliferative ability of TNBC cells (MDA-MB-468, BT549). **B** In vivo mouse xenograft experiments demonstrated the tumor growth of mice injected with circPDCD11-silenced TNBC cells (MDA-MB-468). The data are presented as the mean ± SD. Asterisks indicate significant differences (**p < 0.01).

Moreover, we speculated that circPDCD11 might regulate other phenotypes of TNBC cells. To investigate the role of circPDCD11 in cell proliferation, EdU assays were used, which showed that circPDCD11 knockdown repressed the proliferative ability of TNBC cells and that circPDCD11 overexpression promoted proliferation (Fig. 3A). In vivo mouse xenograft experiments suggested that circPDCD11 knockdown inhibited tumor growth (Fig. 3B). The above results suggested that circPDCD11 enhanced proliferation and that circPDCD11 silencing inhibited tumor growth.

### circPDCD11 targeted miR-432-5p

**Fig. 4:**
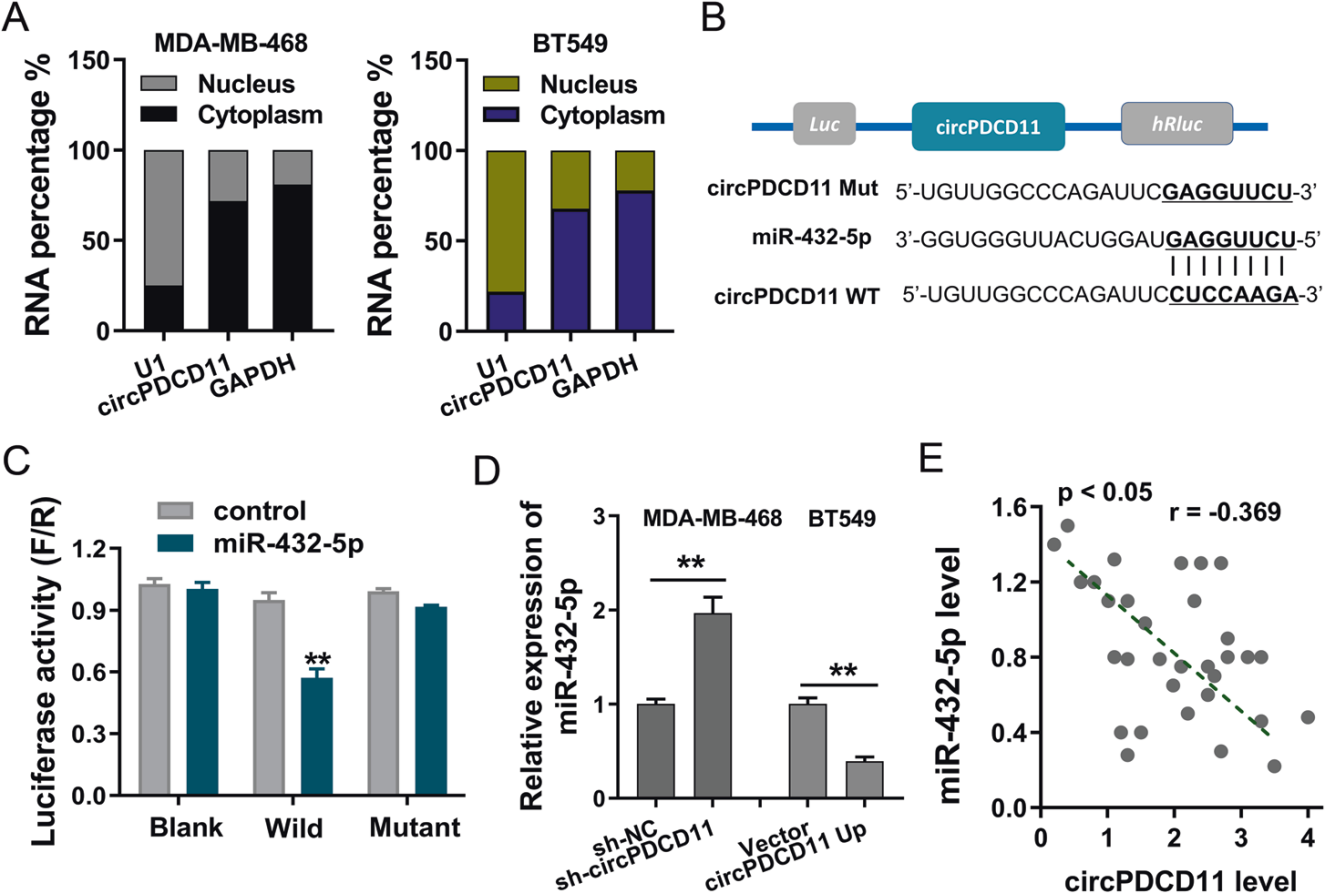
circPDCD11 was identified to target miR-432-5p. **A** The nuclear and cytoplasmic fractions of circPDCD11 were assessed using RT-qPCR in TNBC cells (MDA-MB-468, BT549). **B** The downstream target element of circPDCD11 was predicted using CircInteractome (https://circinteractome.nia.nih.gov/), which revealed that miR-432-5p displayed the complementary binding sites for circPDCD11. **C** A luciferase reporter assay demonstrated the intermolecular interaction (firefly/Renilla) in 293 T cells transfected with circPDCD11 wild type or mutant and miR-432-5p mimics or controls. **D** RT-PCR demonstrated the expression of miR-432-5p in MDA-MB-468 cells transfected with a circPDCD11 knockdown (sh-circPDCD11) and in BT549 cells overexpressing circPDCD11 (circPDCD11 up). **E** Clinical correlation analysis calculated by Pearson’s correlation reflected the correlation between circPDCD11 and miR-432-5p in clinical samples (32 samples). The data are presented as the means ± SD. Asterisks indicate significant differences (**p < 0.01).

To investigate the potential role of circPDCD11, enrichment in the nuclear and cytoplasmic fractions was assessed in TNBC cells (MDA-MB-468 and BT549) (Fig. 4A). The results revealed that circPDCD11 was mainly located in the cytoplasm. Subsequently, the downstream target element of circPDCD11 was predicted using CircInteractome (https://circinteractome.nia.nih.gov/), which found that miR-432-5p displayed the complementary binding sites for circPDCD11 (Fig. 4B). A luciferase reporter assay showed that miR-432-5p could closely interact with circPDCD11 through intermolecular forces (Fig. 4C). Then, RT-qPCR results demonstrated that circPDCD11 knockdown enhanced the expression of miR-432-5p in MDA-MB-468 cells and that circPDCD11 overexpression repressed the expression of miR-432-5p in BT549 cells (Fig. 4D). Clinical correlation analysis found that miR-432-5p was negatively correlated with the expression of circPDCD11 (Fig. 4E). Considering these above results, we concluded that circPDCD11 targeted miR-432-5p.

### LDHA was identified to be targeted by circPDCD11/miR-432-5p

**Fig. 5:**
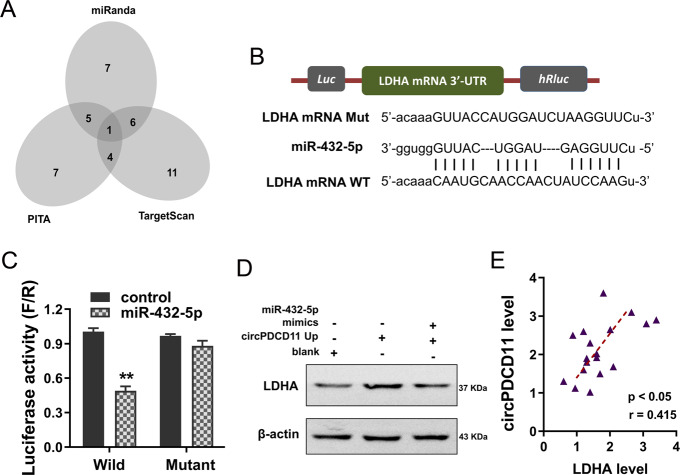
LDHA was identified to be targeted by circPDCD11/miR-432-5p. **A** Online bioinformatics predictive tools identified the potential targets of circPDCD11/miR-432-5p. **B** The multiple complementary binding sites within miR-432-5p and LDHA, including wild type and mutant. **C** The luciferase reporter assay demonstrated the close interaction between wild-type LDHA mRNA 3′-UTR and miR-432-5p. The fluorescence intensity was assessed. **D** Western blot analysis assessed the LDHA protein level in BT549 cells transfected with circPDCD11 overexpression and/or cotransfected with miR-432-5p mimics. **E** Clinical correlation analysis calculated by Pearson’s correlation showed the correlation between circPDCD11 and LDHA. The data are presented as the means ± SD. Asterisks indicate significant differences (**p < 0.01).

In terms of competing endogenous RNAs (ceRNAs), circRNAs frequently regulate proteins by sponging miRNAs. Thus, we investigated the downstream target of circPDCD11/miR-432-5p. Using online bioinformatics predictive tools, we found that LDHA might be as a target of circPDCD11/miR-432-5p (Fig. 5A). There were multiple complementary binding sites within miR-432-5p and LDHA (Fig. 5B). A luciferase reporter assay showed that miR-432-5p could closely interact with the wild-type LDHA mRNA 3′-UTR (Fig. 5C). Western blot analysis found that circPDCD11 overexpression promoted LDHA protein levels, while cotransfection with miR-432-5p mimics alleviated this change (Fig. 5D). Moreover, clinical correlation analysis found that LDHA was positively correlated with the expression of circPDCD11 (Fig. 5E). Considering these above results, we concluded that LDHA was targeted by circPDCD11/miR-432-5p.

## Discussion

An increasing number of studies have demonstrated that the dysregulation of epigenetic modifications displays common features of human cancers. Apart from DNA methylation and histone modification, circRNAs have been recently proposed as a critical regulator in TNBC epigenetic regulation, including cellular apoptosis, protein translation, and RNA splicing [11–13]. In TNBC, the Warburg effect contributes to pathogenesis, metastasis, and recruitment. In the present research, we focused on the potential role of circRNA circPDCD11 in TNBC aerobic glycolysis.

Here, using circRNA microarray analysis, we found that a novel circRNA, circPDCD11, was significantly upregulated in TNBC tissue and cells. Moreover, clinical investigation demonstrated that the high expression of circPDCD11 was associated with a poor prognosis and acted as an independent risk factor for survival. In functional assays, circPDCD11 silencing suppressed proliferation and glycolytic capacity in vitro. In addition, plasmid-mediated circPDCD11 overexpression markedly enhanced proliferation and glycolytic capacity. In vivo assays showed that circPDCD11 silencing inhibited tumor growth. Taken together, these data suggested that circPDCD11 might exert an oncogenic function in TNBC tumorigenesis. An increasing number of studies have shown the critical roles of circRNAs in cancer energy metabolism [14–16]. Importantly, our preliminary results illustrated that circPDCD11 could regulate aerobic glycolysis in TNBC cells; thus, we focused on the potential function of circPDCD11 in TNBC.

The cellular localization determines the function of circRNAs [17]. Generally, a cytoplasmic location tends to indicate posttranscriptional regulation, e.g., miRNA sponging. In addition, a nuclear location tends to indicate transcriptional regulation, e.g., transcription promotion or transcription inhibition. Thus, we first assessed the cellular localization of circPDCD11. The results showed that the cytoplasmic location of circPDCD11 indicated a potential regulation by miRNA sponging. The results of the functional and mechanistic experiments revealed the circPDCD11/miR-432-5p/LDHA axis in TNBC. A luciferase reporter assay found that circPDCD11 could bind to miR-432-5p through covalent binding, and that miR-432-5p also bound to the 3′-UTR of LDHA mRNA. Correlation analysis demonstrated that miR-432-5p was negatively correlated with both circPDCD11 and LDHA mRNA, which was a typical competing endogenous RNA (ceRNA).

CircRNAs are a group of noncoding RNAs without protein-coding potential. In TNBC, an increasing number of circRNAs have been identified to regulate cancer progression [18–21]. For example, circAGFG1 is evidently upregulated in TNBC, and its high level is correlated with the pathological grade, clinical stage, and a poor prognosis. Moreover, circAGFG1 functions as a miR-195-5p sponge to increase cyclin E1 (CCNE1) expression, thereby promoting TNBC progression [22]. CircAHNAK1 is significantly downregulated in TNBC, and its expression is negatively associated with recurrence-free survival (RFS) and overall survival (OS) by modulating miR-421 and RASA1 [22]. Overexpression of circ-ITCH significantly inhibits the proliferation, invasion, and metastasis of TNBC cells in vitro and in vivo. Mechanistically, circ-ITCH acts as a miR-214 and miR-17 sponge to increase its corresponding ITCH linear isoform expression [23]. Collectively, these data indicated that circRNAs could remarkably regulate the pathophysiological process of TNBC [24–26].

**Fig. 6:**
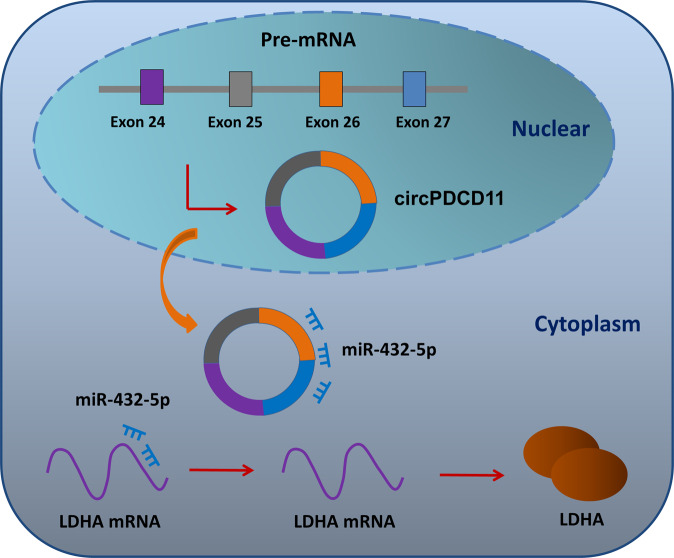
The circPDCD11/miR-432-5p/LDHA axis accelerated aerobic glycolysis in TNBC cells.

TNBC is known for its aggressive phenotype with limited therapies and poor prognosis. Aerobic glycolysis acts as an important stimulatory element and pathological feature for TNBC. Therefore, many oncogenic circRNAs and anticancer circRNAs have been identified using functional experiments to unveil their potential functions. In contrast, identifying circRNAs’ regulatory molecules allows for the identification of their physiological roles. For example, microRNA let-7a-5p targets GLUT12 to regulate TNBC cells’ ATP generation, glucose uptake, lactate production, ECAR, and oxygen-consumption rate [27]. MiR-210-3p targets GPD1L to maintain the stabilization of HIF-1α and inhibits the activity of p53 through CYGB [28]. Tipifarnib-induced decreases in HIF-1α expression are closely associated with the diminution of the Warburg effect, which contributes to the invasion and recurrence of TNBC [29]. In the present research, we found that circPDCD11 promotes aerobic glycolysis in TNBC through the miR-432-5p/LDHA axis (Fig. 6).

In summary, we revealed an important role of novel circPDCD11 in regulating TNBC aerobic glycolysis. The expression of circPDCD11 was upregulated in TNBC and exerted its roles through the miR-432-5p/LDHA axis. More importantly, our findings provide a novel therapeutic strategy to enhance the treatment of TNBC.

## Materials and methods

### TNBC tissue collection

A total of 32 TNBC tissues and the corresponding nontumor tissues were collected in the current study at Peking Union Medical College, which was approved by the Ethics Committee (No. 20117H017). Tissue specimens were histopathologically and clinically diagnosed by pathologists and stored at −80 °C.

### Cell lines

TNBC cell lines (BT549, MDA-MB-468, and MDA-MB-231) and human normal breast cells (MCF10A) were purchased from ATCC (Manassas, VA, USA) and cultured in high-glucose Dulbecco’s modified Eagle medium (DMEM) supplemented with 10% fetal bovine serum, 100 g/ml streptomycin, and 100 U/ml penicillin at 37 °C in humidified air containing 5% carbon dioxide.

### Plasmid constructions and transfection

Stable knockdown of target circPDCD11 was accomplished via lentiviral-based specific short-hairpin RNA (shRNA) by GeneChem (Shanghai, China). In addition, full-length circPDCD11 cDNA was cloned into the vector pLCDH-ciR to overexpress circPDCD11 by RiboBio (Guangzhou, China). Mimics for miR432-5p and siRNAs were synthesized by RiboBio. All oligonucleotides were confirmed by sequencing. Transient transfections were conducted using Lipofectamine 2000 (Invitrogen, NY, USA). The primers are listed in supplementary Table S1.

### Quantitative real-time PCR

Total RNA was isolated from TNBC cells using TRIzol (Invitrogen, USA) reagent following the manufacturer’s instructions. Then, cDNA was synthesized using a portion of the isolated RNA with a PrimeScript RT Master Mix Kit (TaKaRa, Japan). Quantitative real-time PCR was performed using a SYBR Green PCR Kit (TaKaRa, Dalian, China) on an Applied Biosystems 7300 instrument. GAPDH served as the internal control, and the relative expression level of mRNA was calculated using the threshold cycle method (2^−△△Ct^). All primers used in the present study are listed in Table S1.

### Microarray analysis

CircRNA microarray analysis was performed as previously described [30]. Total RNA was extracted from three cervical cancer tissues and three adjacent tissues and then digested with RNase R to remove linear RNA. Microarray procedures and data analysis were performed by the Aksomics Corporation (Shanghai, China). Labeled RNAs were hybridized using an Arraystar Human circRNA Array (8×15 K, Arraystar) (Rockville, USA).

### Western blotting

Total cellular protein was harvested by lysing TNBC cells with RIPA lysis buffer supplemented with proteinase inhibitor cocktail. Protein aliquots were resolved via SDS-PAGE (10%) and then electrotransferred onto polyvinylidene fluoride (PVDF) membranes. PVDF membranes were incubated with primary antibody (anti-LDHA, Abcam, ab52488, 1:1000), and the complexes were detected with enhanced chemiluminescence (ECL) reagents. Beta-actin was used as an internal control.

### Ethynyl deoxyuridine (EdU)-incorporation assay

TNBC cell viability was detected using an EdU assay. In brief, after transfection with the corresponding shRNA or plasmids, approximately 2 × 10^3^ TNBC cells were incubated with 100 μl of 50 μM EdU per well for 2 h at 37 °C. After culturing, cell viability was assessed using the Click-iTR EdU Kit in accordance with the manufacturer’s instructions, and the cells were stained with DAPI for 10 min and visualized using a fluorescence microscope (Olympus). The viability was calculated based on the ratio of EdU-positive cells to the total DAPI-positive cells (blue cells).

### Glycolysis analysis

The glucose uptake of TNBC cells was calculated using Glucose Uptake Colorimetric Assay Kits (Biovision, USA) in accordance with the manufacturer’s instructions. Lactate production in TNBC cells was detected using a Lactate Colorimetric Assay Kit (Biovision, USA) according to the manufacturer’s protocols.

The ECAR was calculated using a Bioscience XF96 Extracellular Flux Analyzer. The ATP level was measured using the CellTiter-Glo Luminescent Cell Viability Assay (Promega, Madison, WI, USA).

### Actinomycin D and RNase R treatment

To measure RNA stability in TNBC cells, actinomycin D (Act-D, 5 μg/ml, Sigma, catalog #A9415) was added. After incubation, the cells were collected, and RNA was isolated for real-time PCR. Levels of circPDCD11 or PDCD11 mRNA were calculated using RT-PCR.

### RNA isolation from nucleus and cytoplasmic fractions

A PARIS Kit (Life Technologies, CA, USA) was used to separate the nuclear and cytoplasmic fractions of TNBC cells according to the manufacturer’s instructions. GAPDH, circPDCD11, and U1 levels in the cytoplasm and nuclear fraction were assessed using RT-qPCR.

### Luciferase reporter assay

For the luciferase reporter assay, full-length wild-type or mutant circPDVD11 (complementary binding sites) and LDHA mRNA 3′-untranslated region (3′-UTR) sequences were synthesized and cloned into the pmiGLO luciferase vector (Promega). Then, miR-432-5p mimics (RiboBio) or blank control RNAs were cotransfected with the above luciferase vectors into 293 T cells using Lipofectamine 2000 (Invitrogen) according to the manufacturer’s instructions. Forty-eight hours later, a Renilla luciferase assay system (Promega, Madison, WI) was used to quantify Renilla luciferase activity.

### Animal in vivo assays

For the in vivo xenograft model, MDA-MB-468/sh-circPDCD11- and MDA-MB-468/control-transfected cells (5 × 10^6^) were injected subcutaneously into the flanks of Balb/C nude mice (five weeks old, male, 10 mice). There were five mice in each group. Tumor growth (length, width, and weight) was monitored after injection and recorded at the indicated time points. Mice were manipulated and housed according to the criteria outlined in the Guide for the Care and Use of Laboratory Animals. This study was approved by the Research Ethics Committee of Peking Union Medical College.

### Bioinformatics analysis

The interaction between circPDCD11 and miRNA was predicted using CircInteractome (https://circinteractome.nia.nih.gov/). The interaction between miRNA and mRNA was predicted using TargetScan (http://www.targetscan.org/), miRanda (http://www.microrna.org/microrna/), and PITA (http://genie.weizmann.ac.il). Pearson’s coefficient correlation was used for circRNA, miRNA, and mRNA expression correlation analyses.

### Statistical analysis

Statistical analysis was performed using GraphPad Prism 8.0 (GraphPad Software, La Jolla, CA, USA) and SPSS 20.0 (Chicago, IL, USA) software. All data are presented as means with standard deviation (SD). Statistical significance was determined using paired or unpaired Student’s *t*-tests or one-way ANOVA. A *p*-value less than 0.05 was considered to be statistically significant.

## Supplementary information

Table S1

## Data Availability

The authors have no research data to share.
