# Supplementary material for: CircRNA circ-PDCD11 promotes triple-negative breast cancer progression via enhancing aerobic glycolysis
Source: Cell Death Discov. 2021 Aug 21;7:218. doi: 10.1038/s41420-021-00604-y (PMC8380247; doi:10.1038/s41420-021-00604-y)
Supplement: Supplementary file 1 — Table S1 [file 41420_2021_604_MOESM1_ESM.docx]

**Supplement Table 1**. Sequences of shRNA and qRT-PCR primers.

|  | 5’-3’ | |  |
| --- | --- | --- | --- |
| circPDCD11 | | forward, 5’-AGGGGCTATGTAGGGTCCAT-3'  reverse, 5'-AGGGTCTTGGAGGAATCTGG-3’ | |
| LDHA | forward, 5’-ATGGCAACTCTAAAGGATCAGC-3'  reverse, 5'-CCAACCCCAACAACTGTAATCT-3' | |  |
| sh-circPDCD11-1 | 5’-GTGTGTTCTTTCGGTTCTGAA-3’ | |  |
| sh-circPDCD11-2 | 5’-GTTCTTTCGGTTCTGAAGCAT-3’ | |  |
| sh-circPDCD11-3 | 5’-GGTGTGTTCTTTCGGTTCTGA-3’ | |  |
| GAPDH | forward, 5’-GGAGCGAGATCCCTCCAAAAT-3’  reverse, 5’-GGCTGTTGTCATACTTCTCATGG-3’ | |  |
